# Are multiple types of associative memory differently impacted by emotion?

**DOI:** 10.1080/02699931.2023.2279182

**Published:** 2023-11-13

**Authors:** Emilie de Montpellier, Deborah Talmi

**Affiliations:** Department of Psychology, University of Cambridge, Cambridge, UK

**Keywords:** Episodic memory, Emotion, Associative memory, Temporal memory

## Abstract

The effect of emotion on associative memory is still an open question. Our aim was to test whether discrepant findings are due to differential impact of emotion on different types of associative memory or to differences in the way participants encoded stimuli across studies. We examined the effect of negative content on multiple forms of associative memory, using the same encoding task. Two registered experiments were conducted in parallel with random allocation of participants to experiments. Each experiment included 4 encoding blocks, in which participants read a neutral text comprised of 6 paragraphs, which were interleaved with neutral or negative images. Images were controlled for visual properties and semantic similarity. Memory tests included recognition memory, Remember/Know, order memory, temporal source memory and contextual memory. Analyses showed that emotion decreased contextual memory but not order memory or temporal source memory. We also found that temporal source memory and contextual memory were correlated. Recognition accuracy and subjective recollection were not impacted by emotion. In agreement with previous work, participants self-reported a reduced ability to integrate blocks containing negative images with paragraphs. In contrast to our hypothesis, results suggest that emotion does not impact all types of associative memory when stimuli are controlled.

## Introduction

The impact of emotion on associative memory is still under debate. Nonetheless, having a better understanding of emotional effects on associative memory could help improve treatments for some debilitating conditions such as PTSD. The dual representation account suggests that an intensely emotional situation will strengthen memory for the negative content but disrupt hippocampal-dependent associative binding (Bisby & Burgess, 2017; Brewin et al., 2010), leading to an inability to store the emotional event in its spatiotemporal context. This theory is supported by studies with healthy volunteers showing a reduced associative memory for emotional images compared to neutral images (Bisby et al., 2018; Madan et al., 2017). However, it is worth noting that other literature suggests improved associative memory in an emotional situation (Henson et al., 2016; Madan et al., 2020; Mickley Steinmetz et al., 2016) or similar associative memory accuracy for both neutral and emotional conditions (Sharot & Yonelinas, 2008).

Other theories claim that emotional items are strongly bound to their context, suggesting that associative memory is increased in an emotional situation. This account would be consistent with findings of experiments using the Remember/Know paradigm, which show that the emotional items strengthen subjective recollection (Dewhurst & Parry, 2000; Kensinger & Corkin, 2003; Rimmele et al., 2011, 2012), suggesting that they are remembered with additional contextual details compared to neutral items. Such findings appear to align well with Context Maintenance and Retrieval Models (CMR3; Cohen & Kahana, 2019; eCMR; Talmi et al., 2019), which simulate strong binding between emotional items and their context.

Empirical findings are not consistent with either of these theories. Indeed, conflicting results are found on the impact of emotion on temporal memory, a form of associative memory. Studies have analysed temporal memory in terms of memory for the order of the items’ presentation, e.g. by re-presenting studied items and requiring participants to reconstruct lists (Huntjens et al., 2015), whilst other studies have scrutinized temporal source memory, wherein subjects encode items appearing in different blocks and are asked to remember in which block (first, middle, last) each item was seen (Rimmele et al., 2012). Although order and temporal source memory can be seen as two types of memory assessing temporal information on a different scale, there are distinctions between them. Order memory can be based partly or wholly on item-to-item associations, whereas temporal source memory does not demand these associations. Temporal source memory tasks require participants to retrieve information about when a stimulus was presented. While one could answer this question by relying on the association between the cue and the temporal source, without retrieving any additional details, there may well be other solutions. It is possible to use the association between the item and non-temporal details about the context in which it was presented if these contextual details are linked to the temporal source. It is also possible to use the association between the item and another item and output the temporal source of the other item. For example, consider an experiment where three items (A1-A3) are presented at three time points (T1-T3), at which contexts C1–C3 prevail. The participant is then asked for the temporal source of item A2. They may state T2 based on the association A2-T2, or on the association chain A2-C2-T2, or indeed state T1 based on the association chain A2-A1-T1. The fact that order memory tasks ask for item-to-item associations whereas temporal source memory tasks can be solved with different mechanisms makes the two types of memory distinctive. Order memory can be perceived as a type of associative memory which is more intrinsic to items than is temporal source memory. Literature investigating memory for the order in an emotional situation presents conflicting results, with some studies showing enhanced accuracy for emotional images (Schmidt et al., 2011) and other studies finding reduced accuracy (Huntjens et al., 2015; Maddock & Frein, 2009). Regarding temporal source memory, some studies show that it is enhanced in an emotional situation (D’Argembeau & Van der Linden, 2005; Rimmele et al., 2012; Schmidt et al., 2011) whilst other studies find no effect of emotion on this type of associative memory (Koenig & Mecklinger, 2008; Minor & Herzmann, 2019).

The contradictory results between studies of associative memory for paired associates, Remember-Know paradigms, and temporal memory suggest that some types of contexts are strongly bound to emotional items and strongly remembered while other types are more easily forgotten. The discrepancy between the effect of emotion on paired-associate memory and Remember responses could be in line with the object-based theory (Mather, 2007) which suggests that emotion enhances binding of items’ features due to increased attention but impairs or has no impact on associations between emotional items and other items or their context. Nonetheless, the inconsistent effect of emotion on temporal source memory, which appears like an internal feature compared to paired associates, challenges the object-based theory. The aim of the current experiment is to better understand how emotion differently impacts distinctive types of associative memory.

In a study that addressed the possibility of differential effects of emotion on multiple dimensions of context, Rimmele and colleagues (2012) investigated how emotion impacts memory for contexts that are thought to be inherently bound to the target stimulus, according to object-based theory. They used a Remember/Know paradigm combined with temporal source and location memory tests. Results showed enhanced recollection of emotional scenes. Temporal source memory was better for negative images and was also higher for items that were given a Remember response compared to a Know response. Similar results were found when participants were asked to remember the location of the image on the screen. Rimmele and colleagues (2012) also investigated associative memory for colour dots placed in the centre of the images. For this type of memory, a double dissociation was found; even though negative images received more Remember responses, memory for dot colour was worse for emotional images compared to neutral ones. If we assume that most Remember responses for emotional items were based on retrieval of internal item features, an assumption partially supported by subsequent work from the same research group (Mihaylova et al., 2019), the results support the object-based theory. More broadly, these results suggest that some types of associative memory, such as temporal source memory or subjective recollection, are enhanced by emotion and others are disrupted.

A more recent study also examined how emotion affects different types of associative memory. Palombo and colleagues (2020) analysed temporal memory and associative memory in the same paradigm, using neutral clips in which neutral and negative images were inserted. Recognition memory for the images inserted in the clips was better for negative compared to neutral images. Temporal memory was measured by asking participants to judge when the image was shown within the clip by clicking on a timeline, and associative memory consisted of recognising the clip in which the image was embedded by showing screenshots. In the negative condition, participants were less accurate in associative recognition but more accurate for temporal judgments. However, the increased temporal accuracy found for the negative images needs to be judged against what appears to be a temporal bias found for neutral images, such that participants estimated neutral images to occur later than they did. When the precision of the temporal judgment was assessed, no difference between emotion conditions was found. Since emotion impacts subjective time perception (Lake et al., 2016; Tipples, 2011), it is possible that asking participants to replace an event on a timeline may reflect time perception for what happened before and after the event, rather than temporal memory for that specific event.

Overall, there is much contradiction between empirical findings on the effect of emotion on associative memory, and it appears that emotion could have different effects on distinct types of associative memory. The discrepant results could also be due purely to methodological differences across studies. To eliminate methodological differences, studies should examine the question of emotional impact on associative memory within the same experiment. Our study adds to this set of studies by comparing how differently temporal source memory, contextual memory, order memory, and subjective recollection are impacted by emotion. In addition, as several types of associative memory were assessed, the present study also look at the relationship between these types of memory. It is plausible that some types of memory are correlated, such as temporal source memory and contextual memory. Indeed, when one is asked to judge in which block a stimulus previously appeared, does one retrieve some contextual details for this block associated with the stimulus, or does the memory for the stimulus appear vivid and detailed, so one can judge that this stimulus was seen recently thus in the last block?

Our paradigm was designed with the aim of assessing temporal memory in addition to other types of associative memory, therefore it needed a context that unfolds over time to mimic the stream of sensory information changing over time outside the laboratory context, a core feature of the theories that guided our work (CMR3; Cohen & Kahana, 2019; Hintzman, 2016; eCMR; Talmi et al., 2019). As described above, previous paradigms looking at temporal memory examined memory for the experiment blocks that stimuli were embedded in (Rimmele et al., 2012; Schmidt et al., 2011); in these paradigms the stimuli themselves provide the unfolding context. The paradigm designed by Palombo and colleagues (2020) used neutral clips as context and pictures inserted in these clips. We decided not to use clips here, to reduce the amount of visual information participants see, and to use neutral texts instead. In addition, Palombo and colleagues (2020) inserted one image per context; we decided to insert several images (one after each paragraph) per block, to eliminate any surprise effects. Our paradigm was then modified based on the results of a pilot study (not reported here). The pilot study investigated contextual memory and order memory using an encoding task composed of four blocks, with a generic neutral context in each block. The generic context was a text (one topic per block) divided into paragraphs to induce a temporal aspect. In addition, the paradigm included a sequential presentation of images (one image after each paragraph) so that when asked about order memory, time estimation was not involved. The results were close to floor therefore the paradigm was modified to improve performance; order memory was assessed after each block rather than after the whole encoding task, participants were asked to integrate the images and the text together to improve contextual memory performance and the stimuli set was modified. Nonetheless, the structure of the design remained for the main experiment (4 blocks including images interleaved by paragraphs).

**Figure 1.**
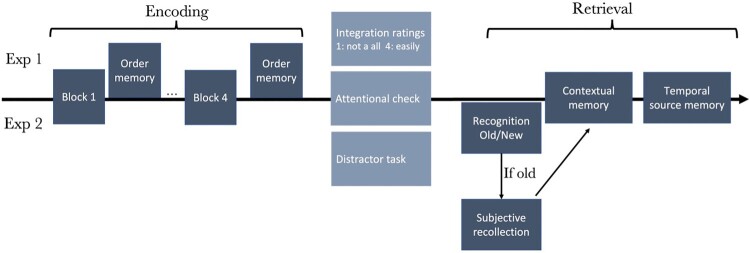
Method of Experiments 1 and 2. Both experiments included a temporal source memory test and a contextual memory test. Experiment 1 included an order memory test after each block and Experiment 2, a recognition memory test and a Remember/Know test after encoding. Between encoding and retrieval, participants had to rate their integration ability, go through an attentional check question and a distractor task.

**Figure 2.**
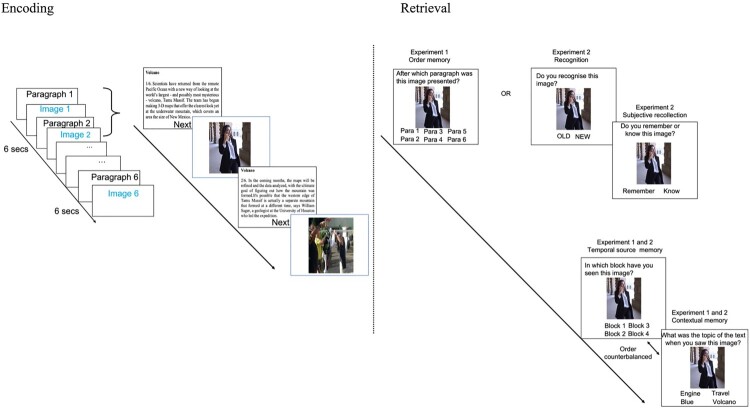
Encoding and Retrieval of Experiments 1 and 2. Encoding was composed of four blocks including 6 paragraphs interleaved by 6 images. The left side of the figure shows an example of neutral block with the first four trials. Retrieval was composed of order, temporal source and contextual memory tests for Experiment 1 and recognition memory, Remember/Know, temporal source and contextual memory tests for Experiment 2. The right side of the figure shows an example of trial for each memory test.

Our experiment reported here aimed at assessing temporal source memory, contextual memory, order memory, and subjective recollection. However, order memory and subjective recollection could not be assessed in the same experiment: order memory had to be assessed after the presentation of a few items (after each block), but by doing so, participants would see each item twice, which could be a confound for subjective recollection results. To overcome this, the experiment was split into two experiments with the same encoding paradigm. Both experiments included a final test of temporal source memory task and a contextual memory task, administered when encoding was completed. The two experiments also included a third task. Experiment 1 included an order memory task, administered at the end of each block, while Experiment 2 included a Remember/Know memory test, administered prior to the temporal source/contextual memory tasks. Participants were randomly allocated to one of the experiments to enable joint analysis of the tasks common to both (see Figures 1 and 2 for the method’s differences between Experiments 1 and 2). The two experiments were pre-registered. The pre-registered hypotheses were that temporal order memory, temporal source memory and the number of Remember responses would be enhanced for emotional items whereas contextual memory would be disrupted.

## Experiment 1

### Method

The method and plan of analysis of the data of Experiment 1 were pre-registered (https://osf.io/bv8wz/?view_only=b5e25e10b23244c4af62abd42a9b7685).

### Participants

Several comparisons were planned which had different effect sizes in previous literature (order memory; D’Argembeau & Van der Linden, 2005; contextual and temporal memory; Palombo et al., 2020; Rimmele et al., 2012), thus, a medium effect size of *d* = 0.5 was used (power = 0.9, *α* = 0.0166). α was adapted for 3 multiple comparisons (significance threshold *p *= 0.0166). Power analysis, conducted with the program G*Power, suggested that a sample of *N* = 57. The initial sample size was 71 participants (52 females; mean age, 28.5 years; SD, 6.16; for demographics details see Appendix: Table A3), 11 failed the attentional check (see below) and 3 reported having encountered technical issues, therefore the final sample used for the analyses was 57 participants, corresponding to the sample size required by the power analysis.

All experiments were approved by the University of Cambridge Psychology Research Ethics Committee (PRE.2021.009). All participants provided written informed consent. Participants were recruited through the recruitment platform “Prolific”. They were reimbursed for their time (£4 for 30 min). All participants were between 18 and 40 years of age and were fluent in English. Exclusion criteria included people who had a diagnosis of mental health disorders, or with medical issues such as perception or motor problems that would make participation challenging, or neurological disorders, such as dyslexia or past concussions as well as people who took medication or recreational Pl drugs that influence brain function (psychoactive drugs).

### Materials

Images: Negative and Neutral images were taken from a previous study dataset (Riberto et al., 2022). The images were controlled for visual features (luminance, contrast, Red, Green, Blue, JPEG size and entropy) and thematic similarity, by only selecting images with an action-context combination and depicting people outdoors. The original study reports that ten healthy participants rated the valence and the arousal of the stimuli. Emotional and neutral pictures were significantly different in valence (*F* = 46.93, *p < *0.001) and in arousal (*F* = 27.37, *p < *0.001), with emotional pictures rated lower in valence, and higher in arousal, than the neutral images. Images included 4 different categories (2 neutral and 2 negative). The 2 neutral categories were people hanging laundry and people on their phones, and the 2 negative categories were poverty scenes and car accident scenes.

Here, 2 stimulus sets were created, set A and set B, each composed of 12 neutral images (6 from the category depicting people hanging laundry and 6 from the category of people on their phone) and 12 negative images (6 images showing poverty scenes and 6 images showing car accidents scenes). Half of the participants saw set A and half saw set B during the encoding. The 24 images were controlled for visual properties and neutral images were rated as significantly different from negative images in valence and arousal (see Appendix: Table A7).

Texts: The texts were selected from two websites (https://www.ielts-exam.net/ielts_reading/; https://ielts-up.com/reading/ielts-reading-practice.html#academic) and were similar in nature to educational texts presented during the International English Language Testing System. The 4 texts were rated by two independent researchers. The overall valence rating was between 4.5 and 6.5 (with 1 being negative and 9 being positive) and the overall arousal rating was between 4 and 6 (with 1 being relaxed and 9 being aroused). Texts included 6 paragraphs. Paragraph length was between 49 and 86 words (*M* = 64.54 words). Total text length was between 367 and 398 words (*M* = 384.5 words). Texts were counterbalanced across emotional and neutral conditions.

### Procedure

The experiment was online, using the platform GORILLA (Anwyl-Irvine et al., 2020). At the end of the experiment, participants were asked whether they took notes during the study or encountered technical issues. If they did, they were excluded from the study. Participants were also excluded if they failed the attentional check by giving more than 1 incorrect answer (out of 4) to the comprehension questions about the text. The 4 questions were each presented after each block that included the text of interest. The questions were easy to reply to and could be answered in one word (ex. Which country was mentioned in this text?).

*Encoding:* Before starting the encoding task, participants read instructions specifying the orienting task which was that they must pay attention to the text and the images and find some associations between them. They reported their success at the end of each block. They were also informed that they would be asked about the order of the presentation of the images. During the task, participants saw 4 blocks, each composed of one text divided into 6 paragraphs. Participants read paragraphs at their own pace and had to press “next” when they were done reading the paragraphs. After each paragraph, one image was presented for 6000 ms (6 images were presented per block). There was no button press when seeing the images. The images were all neutral in one block, all negative in one block and an equal mix of negative and neutral in two blocks. These two mixed blocks contained 3 neutral images from the two categories (phone and laundry) and 3 negative images also from the two categories (car accidents and poverty). The order of the blocks was randomised. The presentation order of the images within blocks was randomised.

At the end of each block, participants (i) rated the extent to which they were able to associate the text and the images (integration ratings) on a 1–4 scale (1 = not at all, 4 = easily) (ii) answered one comprehension question about the text they had just read, which was used as the attentional check (iii) completed a distractor task, composed of five mathematical problems (iv) completed the order task.

*Retrieval:* After each block, participants completed the (i) order task. At the end of the presentation of all 4 blocks, all images were presented one by one, twice, once for the (ii) temporal source memory task, and once for the (iii) contextual memory task (Figure 2). The order of the last two memory tasks was counterbalanced.

- Order task: After each block, participants were presented with the 6 images seen during the block. One image was at the top of the screen, and participants were asked to remember after which paragraph the image was presented, by selecting from 6 listed options: paragraphs 1–6.
- Temporal source memory task: One image was presented at the top of the screen and participants were asked to remember in which block the image was presented, by selecting from 4 listed options: block numbers 1–4.
- Contextual memory task: One image was presented at the top of the screen and participants were asked to remember what the topic of the text was when the image was presented, by selecting from 4 listed options: the four topics of the four blocks.

### Statistical analysis

Order memory, temporal source memory and contextual memory were analysed by measuring the proportion of correct responses. We pre-registered a simple analysis that will first be reported; paired t-tests for order memory, contextual memory, and temporal source memory. For this main analysis *p-values* for the t-tests were corrected using the Holm–Bonferroni method, as stated in the pre-registration. The uncorrected *p-values* and Bayes factors (BF) will also be reported. We also pre-registered the aim to analyse the effects of block-type, and correlations across different types of associative memory, but the pre-registration did not specify the analysis plan. In light of this, we conducted generalized linear mixed effect models across trials. This statistical method allowed us to control for random intercepts for participants. Random slopes were not included due to convergence issues. The models were analysed using a likelihood-ratio test method with the software R with the package lme4 (for more details on the procedure and the packages used: see Brown, 2021), enabling us to deduce which model best suits our data.

### Results

#### Order memory

**Figure 3.**
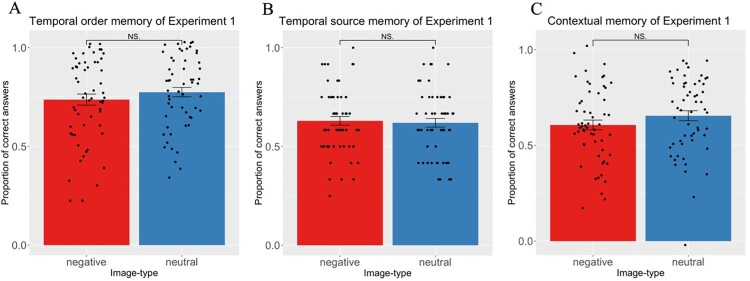
Results of Experiment 1. (A) Order memory. No difference between negative and neutral images was found. (B) Temporal source memory. No effect of emotion was found. (C) Contextual memory. No effect of emotion was found. NS = non significant

No effect of emotion was found for order memory. No difference in memory performance for negative (*M* = 0.736; SD = 0.211) and neutral (*M* = 0.774; SD = 0.18) conditions was found (t(56) = 1.580, *p *= 0.12, *p(Holm–Bonferroni) *= 0.28*, d = 0.209*; BF = 2.89; Figure 3A).

The model Order_memory ∼ contextual_memory + temporal source_memory + emotion + block-type + contextual_memory*emotion + temporal source_memory *emotion + (1| participant) was generated. No significant predictors were found (*p*’s > 0.063). For details regarding model parameters, see Appendix: Table A4.

Additionally, as pre-registered in the exploratory analysis, biases were measured to investigate whether participants were more likely to remember a neutral or negative image as appearing later or earlier than its actual occurrence. To compute bias, the response for each image for each participant was subtracted from the actual occurrence of the image. For instance, if the participant answered, “after block 4” and the image was presented after block 2, the bias score was +2. Negative scores would indicate that the temporal judgment bias was earlier than the actual occurrence and positive scores would show a later bias than the actual occurrence. The average score across participants for negative and neutral images was then computed. No difference was found between the two conditions (neutral; *M* = −0.0146; SD = 0.18; negative; *M* = 0.117; SD = 0.1976; t(56) = −0.769, *p *= 0.445*; d = −0.1*).

The precision of temporal order judgments was also measured. To compute precision, the absolute value of the above bias scores was computed. The further the score is from 0 (indicating that the participant gave the correct answer), the further the answer is from the actual occurrence of the image. The average score across participants for negative and neutral images was then computed. No difference was found between the two conditions (neutral; *M* = 0.3304; SD = 0.2834; negative; *M* = 0.3655; SD = 0.3347; t(56) = −0.765*, p *= 0.447*; d = −0.1*).

#### Temporal source memory

No difference between temporal source memory was found between the negative (*M* = 0.63; SD = 0.166) and neutral (*M* = 0.619; SD = 0.165) conditions (t(56) = 0.45, *p *= 0.653*, p(Holm–Bonferroni) *= 0.653; *d = −0.06;* BF = 8.705; Figure 3B).

The model: Temporal source_memory ∼ order_ memory + contextual_memory + emotion + block-type + order_memory*emotion + contextual_memory*emotion + (1| participant) was generated. The likelihood-ratio test indicated that models including contextual memory (χ2(1) = 103.30, *p *< 0.001), block-type (χ2(1) = 12.88, *p *< 0.001), and the interaction between emotion and contextual memory (χ2(1) = 15.03, *p *< 0.001) as predictors were best fits to the data (for details regarding model parameters, see Appendix: Table A4).

#### Contextual memory

No difference was found in the contextual memory performance for the negative (*M* = 0.606; SD = 0.192) and neutral (*M* = 0.654; SD = 0.18) conditions (t(56) = 1.71, *p *= 0.092, *p(Holm–Bonferroni) *= 0.28*; d = 0.23;* BF = 2.34; Figure 3C).

The model Contextual_memory ∼ order_memory + temporal source_memory + emotion + block-type + order_ memory*emotion + temporal source_memory* emotion + (1| participant) was generated. The likelihood-ratio test indicates that order memory (χ2(1) = 4.12, *p *= 0.042), temporal source memory (χ2(1) = 98.38, *p *< 0.001), block-type (χ2(1) = 6.77, *p *= 0.009), as well as the interaction between emotion and temporal source memory (χ2(1) = 15.91, *p *< 0.001) were significant predictors. For details regarding model parameters, see Appendix, Table A4.

#### Exploratory analysis: integration ratings

We pre-registered an exploratory analysis of the impact of emotion on integration ratings. Paired t-tests indicated that integration ratings were lower for pure negative blocks (*M* = 1.61; SD = 0.773) compared to pure neutral (*M* = 1.94; SD = 0.83; t(56) = 2.59*, p *= 0.012, *p(Holm–Bonferroni) *= 0.04*; d = 0.34*). No other significant differences were found (all *p*’s*(Holm–Bonferroni) > *0.058).

### Discussion

Experiment 1 examined the effect of emotion on three different types of memory: order memory, contextual and temporal memory. The results showed that none of the associative memory tested were impacted by emotion. These findings contrast with the literature; contextual memory was shown to be reduced in an emotional situation while temporal source and order memory appeared increased (Bisby et al., 2018; D’Argembeau & Van der Linden, 2005; Madan et al., 2017; Rimmele et al., 2012; Schmidt et al., 2011 but see: Huntjens et al., 2015; Maddock & Frein, 2009). An additional associative memory supposedly affected by emotion is subjective recollection. Since order memory was tested after each block, subjective recollection could not be assessed in Experiment 1. Experiment 2 was therefore conducted to analyse subjective recollection, temporal source and contextual memory.

## Experiment 2

### Method

The method was similar to Experiment 1 other than the following differences.

The method and plan of analysis of the data of Experiment 2 were pre-registered (https://osf.io/qm4z9/?view_only=fd74558fec564a7c9fcc5fd2617ace90). Deviations from the pre-registered analyses are noted below. Experiment 2 was run at the same time as Experiment 1 and participants were randomly allocated to one of the two experiments.

#### Participants

Similar to Experiment 1, several comparisons were planned which had different effect sizes in previous literature (contextual and temporal memory; Palombo et al., 2020; subjective recollection, contextual and temporal memory; Rimmele et al., 2012), therefore, a medium effect size of *d* = 0.5 was used (power = 0.9, *α* = 0.01). α was adapted for 5 multiple comparisons (significance threshold *p = *0.01). Power analysis, conducted with the program G*Power, suggested that a sample of *N* = 63. The initial sample size was 87 participants (62 females, 1 other, 1 participant did not report their gender, mean age, 27.2 years; SD, 5.66; for demographics details see Appendix: Table A3). 17 participants failed the attentional checks, 1 participant reported taking notes, 2 participants reported taking notes and having encountered technical issues and 4 participants reported having encountered technical issues, therefore the final sample used for the analyses was 63 participants, corresponding to the sample size required by the power analysis. Similar exclusion criteria were used as in Experiment 1.

#### Materials

Similar to Experiment 1, except that, for this experiment, the two sets of images (A and B) were used for all participants and the images from the unseen set were used as lures in the recognition task.

#### Procedure

Compared to Experiment 1, the order task at the end of each block was eliminated (see Figures 1 and 2 for the method’s differences between Experiments 1 and 2); recognition memory and Remember/Know tasks were inserted before the temporal source memory and contextual memory tasks. The order task was removed because it could have been a confound for subjective recollection, as participants would have seen the images twice (during the encoding and order task) before being asked about subjective recollection.

*Encoding:* Similar to Experiment 1 without the order task.

*Retrieval:* Compared to Experiment 1 the order of the memory tests is different; for Experiment 2, participants saw one image, were asked about recognition of this image (OLD or NEW), Remember/Know question, justification of their Remember response (if they answered Remember), contextual and temporal memory questions (if they answered OLD). Participants then moved on to the next image and the process was repeated.

- Recognition memory: After the encoding task and a distractor task, one image was presented on the top of the screen and participants were asked to judge whether they had seen this image by pressing one of the two buttons: “OLD” or “NEW”. If the image was categorized as OLD, participants then undertook the Remember/Know task, the contextual memory task and the temporal source memory task. The procedure of the latter two tasks resembled Experiment 1, and as in Experiment 1, their order was counterbalanced. In total, 24 old images and 24 new images were presented.
- Remember/Know judgment: Participants were instructed before the retrieval task about the difference between a Remember judgment and a Know judgment. They were also given examples. The instructions were inspired by the study of Geraci and colleagues (2009). If the image was categorized as “Remembered”, participants were then asked to indicate what detail they could remember from the encoding context, by selecting from 2 listed options: “Details related to the image” and “Details unrelated to the image”.

#### Statistical analysis

Recognition memory was analysed by comparing memory accuracy (Hits minus False alarms) between the negative and the neutral conditions. Additionally, Hits and False alarms between the two conditions were compared. BR index for each condition was also computed according to the Equation 8 in Snodgrass and Corwin (1988): BR = False alarms /1 -(Hits-False alarms) and compared. Remember/Know judgments were analyzed by comparing the proportion of Hits that were “Remembered” minus the proportion of False alarms that were “Remembered” between the negative and the neutral conditions. Justification of the Remember responses was analysed by comparing the number of times participants chose the option “Details related to the image” for Remembered emotional images and for Remembered neutral images. Temporal source memory performance and contextual memory were analysed in a similar way as Experiment 1.

Similar to Experiment 1, we pre-registered a simple analysis (paired t-tests and McNemar test for the Remember justifications) that will first be reported. In addition, the exploratory pre-registered aim to analyse the effects of block-type, and correlations across types of associative memory was achieved using mixed effect models analysing Remember/Know judgement, temporal source memory and contextual memory, which will be reported. Similar to Experiment 1, mixed effect models included random intercepts to control for participants but not random slopes, due to convergence issues.

### Results

#### Recognition memory

**Figure 4.**
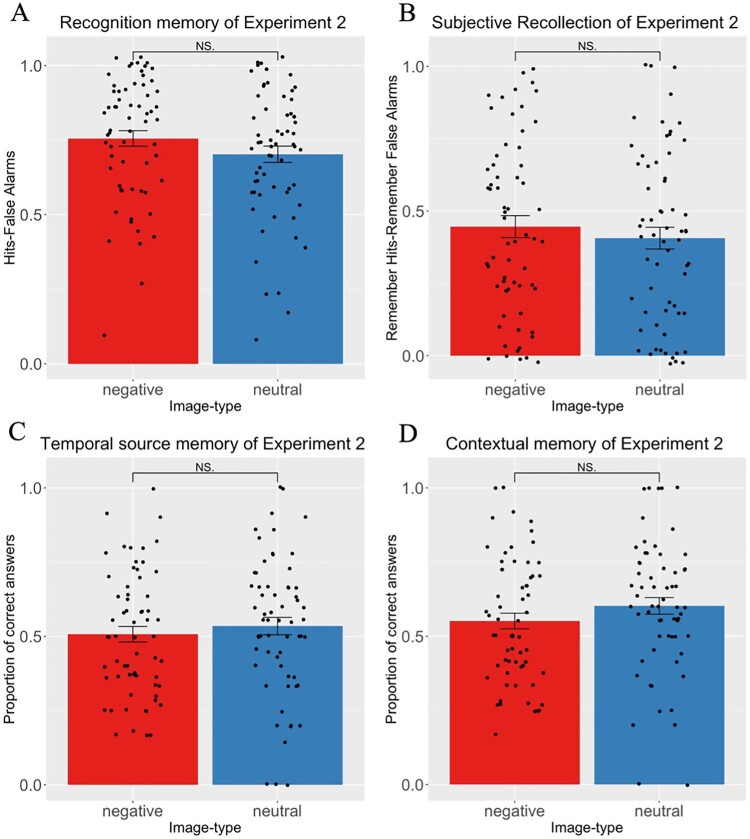
Results of Experiment 2. (A) Recognition accuracy. No difference between negative and neutral images when the *p*-value was corrected for Holm-Bonferroni. (B) Subjective recollection. No effect of emotion was found. (C) Temporal source memory. No effect of emotion was found. (D) Contextual memory. No effect of emotion was found. These results replicate the findings of Experiment 1. NS = non-significant.

A trend was found showing better recognition memory for negative images (*M* = 0.755; SD = 0.207) compared to neutral images (*M* = 0.702; SD = 0.216; t(62) = 2.056, *p *= 0.044; *d = −0.26;* BF = 1.34; Figure 4A), but the *p*-value did not survive Holm–Bonferroni corrections: *p(Holm–Bonferroni) *= 0.17. Further analyses showed higher proportion of Hits for the negative condition (*M* = 0.794; SD = 0.203) compared to the neutral condition (*M* = 0.726; SD = 0.2, t(62) = 2.838, *p *= 0.006, *d *= −0.36). No difference in the proportion of False alarms between the two conditions was found (neutral; *M* = 0.024; SD = 0.062; negative; *M* = 0.038; SD = 0.066; t(62) = 1.332, *p *= 0.188, *d *= −0.166). A BR equal to 0.5 indicates a neutral bias, a BR above 0.5 indicates a liberal bias and below 0.5, a conservative bias (Snodgrass & Corwin, 1988). BR indices indicated that participants had a conservative bias for both conditions (neutral; BR = 0.05; SD = 0.129; negative; BR = 0.16; SD = 0.29), but a significant difference between the two conditions suggested that participants were more liberal in the negative condition (t(62) = 2.753, *p *= 0.008, *d *= −0.34).

#### Subjective recollection

No difference was found between the proportion of Remember responses for negative images (*M* = 0.445; SD = 0.301) and neutral images (*M* = 0.406; SD = 0.229; t(62) = 1.54, *p *= 0.128*, p(Holm–Bonferroni) *= 0.38; *d = 0.19;* BF = 3.18; Figure 4B).

The model Remember responses ∼ contextual_ memory + temporal_memory + emotion + block-type + contextual_memory*emotion + temporal_memory* emotion + (1| participant) was generated. The likelihood-ratio test indicated that the model including block-type (χ2(1) = 5.13, *p *= 0.023) as a predictor provides a better fit for the data than a model without it (see Appendix: Table A5).

#### Justification of the remember judgment

There was a modification from the pre-registration for the analysis of the results for the justification of Remember responses, using a McNemar test instead of a chi-squared test. It appeared more adequate as our data were paired and repeated measures. The test showed no difference between the proportion of answers “related to the image” between negative (0.396) and neutral images (0.509; *p *= 0.134: two-sided).

#### Temporal source memory

No difference in the temporal source memory performance for the negative (*M* = 0.507; SD = 0.207) and neutral (*M* = 0.534; SD = 0.232) conditions was found (t(62) = 0.794, *p *= 0.429*, p(Holm–Bonferroni) *= 0.429; *d = 0.1,* BF = 7.409; Figure 4C).

The model: Temporal source memory ∼ remember_responses + contextual_memory + emotion + block-type + remember_responses*emotion + contextual_memory *emotion + (1| participant) was generated. The likelihood-ratio test indicated that the model including contextual memory (χ2(1) = 536.35, *p < *0.001) provides a better fit for the data than a model without it. For details regarding model parameters, see Appendix: Table A5.

#### Contextual memory

No difference was found in the memory performance for the neutral (*M* = 0.602; SD = 0.223) and negative (*M* = 0.551; SD = 0.208) conditions (t(62) = 1.49, *p *= 0.139*, p(Holm–Bonferroni) *= 0.38*; d = 0.19;* BF = 3.409; Figure 4D).

The model: Contextual_memory ∼ remember_ responses + temporal source_memory + emotion + block-type + remember_responses*emotion + temporal source_ memory*emotion + (1| participant) was generated. The likelihood-ratio test indicated that temporal source memory (χ2(1) = 537.40, *p *< 0.001), block-type (χ2(1) = 17.30, *p *< 0.001), as well as the interaction between emotion and temporal source (χ2(1) = 4.45, *p *= 0.035) were significant predictors. For details regarding model parameters, see Appendix: Table A5.

#### Integration ratings

Similar to Experiment 1, paired t-tests indicated that integration ratings were lower for pure negative blocks (*M* = 1.46; SD = 0.7144) compared to pure neutral (*M* = 2.11; SD = 1.033; t(62) = 4.718*, p *< 0.001, *p(Holm–Bonferroni) *< 0.001; *d = 0.59*). In contrast to Experiment 1, a difference between mixed (*M* = 1.706; SD = 0.699) and pure neutral blocks was found (t(62) = 2.838*, p *= 0.006*, p(Holm–Bonferroni) *= 0.012; *d = −0.36*), showing lower ratings for mixed compared to pure neutral blocks. A difference between mixed and pure negative blocks was also found (t(62) = 2.32*, p = *0.024*, p(Holm–Bonferroni) = *0.024; *d = 0.29*), showing higher ratings for mixed compared to pure negative blocks.

### Discussion

Experiment 2 examined the effect of emotion on subjective recollection, contextual and temporal source memory as well as recognition memory. Emotion had no effect on memory assessed and this contrasts with previous literature (Bisby et al., 2018; Choi et al., 2013; D’Argembeau & Van der Linden, 2005; Madan et al., 2017; Phelps, 2006; Phelps & LeDoux, 2005; Rimmele et al., 2012; Schmidt et al., 2011). Interestingly Experiment 2, similar to Experiment 1, showed that contextual memory and temporal memory were correlated. Both tests may be measuring similar mechanisms. Lastly, integration ability was consistently rated as lower when negative images were included in blocks. To increase power, data of Experiments 1 and 2 were joined to analyse temporal source and contextual memory as well as integration ratings.

## Experiments 1 and 2

**Figure 5.**
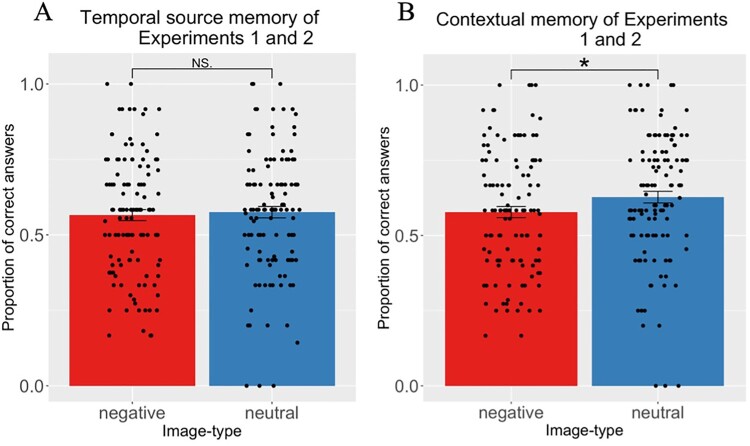
Results of Experiments 1 and 2 combined. (A) Temporal source memory. No effect of emotion. (B) Contextual memory. Reduction of contextual memory for negative images. NS = non-significant, * = *p* < 0.05.

**Figure 6.**
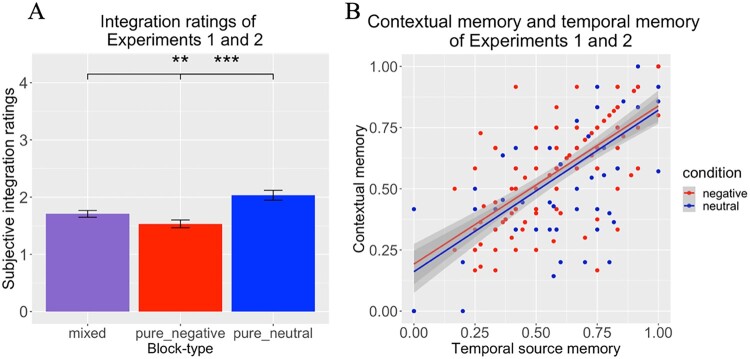
(A) Integration ratings results for Experiments 1 and 2 combined. Results showed reduced integration ratings for pure negative blocks compared to mixed blocks, for pure negative compared to pure neutral and for mixed compared to pure neutral. (B) Figure showing the significant correlation between contextual memory performance and temporal source memory for both conditions (emotional and neutral), as reported by the mixed effect models. Neutral r(120) = .677, *p* < .001, Negative r(120) = .658, *p* < .001. * = *p* < 0.05; ** = *p* < 0.01; *** = *p* < 0.001.

Mixed effect models were run across trials for temporal source memory and contextual memory on the data of Experiments 1 and 2 joint (Figure 5; Figure 6B). ANOVAs were conducted for the analyses of integration ratings (Figure 6A). These analyses were not pre-registered.

### Results

#### Temporal source memory

The model was: Temporal source_memory ∼ contextual_memory + emotion + block-type + contextual_memory*emotion + (1| participant). The likelihood-ratio test indicated that contextual memory (χ2(1) = 530.11, *p *< 0.001; Figure 6B) was a significant predictor. For details regarding model parameters, see Appendix: Table A6.

#### Contextual memory

The second model was: Contextual_memory ∼ temporal source_memory + emotion + block-type + temporal source_memory*emotion + (1| participant). The likelihood-ratio test indicated that temporal source memory (χ2(1) = 527.83, *p *< 0.001), emotion (χ2(1) = 10.52, *p *= 0.001), as well as block-type (χ2(1) = 14.61, *p *< 0.001) were significant predictors (Figure 5 & Figure 6B). For details regarding model parameters, see Appendix: Table A6.

#### Integration ratings

Overall mean: 1.75 on a 1–4 scale. To assess how block-type impacts the ability to integrate the images and the context together, a 3 × 2 ANOVA was conducted. The dependent variable was integration ratings, the between-subject factor was Experiment (1 or 2), and the within-subject factor was Block-type (mixed; pure-negative; pure-neutral). An effect of Block-type was found (F(2, 118) = 16.416, *p *< 0.001*, η_p_^2 ^= 0.122*), but no effect of Experiment (F(2, 118) = 0, *p *= 0.985, *η_p_^2 ^= 0.908*), nor an interaction Block-type* Experiment (F(2, 118) = 1.659, *p *= 0.194, *η_p_^2 ^= 0.014*) were found. For the Block-type effect, pairwise comparisons (Bonferroni corrections) indicated lower ratings for mixed blocks compared to pure neutral (t(119) = 3.6*, p *= 0.002*, d = 0.329*), lower ratings for pure negative compared to pure neutral (t(119) = 5.24, *p *< 0.001, *d = 0.478*) but no difference between mixed and pure negative (t(119) = 2.32, *p *= 0.074*, d = 0.212;* Figure 6A).

## General discussion

The primary aim of this study was to investigate whether different types of associative memory are differently impacted by emotion when stimuli are controlled. We examined the effect of emotion on order memory, contextual memory, temporal source memory, recognition memory, and subjective recollection. For practical reasons, we separated these tests into two very similar experiments, which were conducted simultaneously, with random allocation of participants to experiments. Encoding varied very little between experiments and tests, the main difference being the inclusion of an order memory test after each block in Experiment 1. Despite above-chance performance on all tests, we did not observe an effect of emotion on any of the associative memory tests in the pre-registered tests. The only effect of emotion we observed was on contextual memory, where emotion decreased contextual memory in the mixed effect model analysis that combined data from the two experiments. A second aim of the study was to explore how the various measures of associative memory were related to each other. Mixed effect models revealed that temporal source memory and contextual memory were significantly related. One last aim was to examine how emotion influences the integration of emotional content and non-emotional context. Interestingly, we found that integration ratings were lower for blocks including a negative image.

The results showing that emotion decreased contextual memory when data from Experiments 1 and 2 were combined is in line with the dual representation theory, which argues that there is a reduction in associative memory in an emotional situation (Brewin et al., 2010) and with our pre-registered hypothesis. However, we should be cautious in interpreting this result since the effect was not found in the pre-registered analyses when looking at Experiments 1 and 2 separately. Additionally, although there are some reports of decreased contextual or paired-associate memory in an emotional situation (Bisby et al., 2018; Bisby & Burgess, 2013; Madan et al., 2012, 2017; Palombo et al., 2020), some studies reported no effect of emotion on contextual memory (Sharot & Yonelinas, 2008) or enhanced contextual memory for emotional situations (Henson et al., 2016; Madan et al., 2020; Mickley Steinmetz et al., 2016). Nonetheless, this result still suggests that memory for the context may be reduced in an emotional situation.

In contrast to our hypotheses, no emotional effect was seen on either test of temporal memory. Experiment 1 shows that when images are interleaved by paragraphs reflecting a generic context, and participants are asked to remember their initial order, there is no difference between memory for the order of emotional and neutral images. These results contrast with some reports where temporal order for emotional situations was enhanced (Schmidt et al., 2011) or reduced (Huntjens et al., 2015; Maddock & Frein, 2009). Nonetheless one study found no relationship between emotion and order memory (Makowski et al., 2017). It is worth noting that intentional instructions were used for the order memory task to avoid block 1 being processed differently from blocks 2–4, as memory was tested after each block. Given our design, it was necessary to probe intentional order memory, but one should note that order memory may have been differently impacted by emotion in an incidental setting. Temporal source memory was also not influenced by emotion either in Experiment 1 or 2. These results contrast with the study of Rimmele and colleagues (2012), who found an enhanced temporal source for emotional items. Yet other findings showed that temporal source memory is not impacted by emotion (Koenig & Mecklinger, 2008; Minor & Herzmann, 2019). In these studies, intentional encoding instructions were given, which was suggested as a potential cause of null findings (Petrucci & Palombo, 2021). However, in the present experiment participants were not aware that they would be asked to retrieve the block in which each image was presented. Therefore, intentional encoding cannot be the explanation for our findings. Participants in Experiment 1 were asked to intentionally consider image order, which may have contributed to the null results; but in Experiment 2 they did not, and the results replicated those in Experiment 1. Taken together, our results suggest that emotion does not robustly attenuate temporal memory.

More broadly, it is worth noting that Experiment 1 probes intentional memory whereas Experiment 2 probes incidental memory. For Experiment 1, it is possible that participants have processed the stimuli during encoding to perform a memory test as they were aware that their order memory would be tested after each block. Intentional and incidental memory are known to be different regarding the level of processing they are triggering (Craik & Lockhart, 1972). Experiment 2 was purely incidental. Previous literature showed that recall and recognition are differently impacted by intentional versus incidental instructions (Eagle & Leiter, 1964; Estes & Da Polito, 1967). In addition, a more recent study found that intentionality may impact episodic memory via increased processing of context-item associations, as the study reveals that contextual memory was increased for the to-be-remembered items compared to the items in the incidental condition (Popov & Dames, 2023). In the current study, although necessary for the design, the different instructions could be considered as a caveat for the joint analysis of Experiments 1 and 2. The general increased accuracy in Experiment 1 compared to Experiment 2 (Contextual memory M_exp1 _= 0.63; Contextual memory M_exp2 _= 0.58; Temporal source memory M_exp1 _= 0.62; Temporal source memory M_exp2 _= 0.52) may reflect instruction differences.

Recognition memory was overall not impacted by the emotionality of the images, in contrast to much previous literature (Choi et al., 2013; Phelps, 2006; Phelps & LeDoux, 2005). While this effect was not seen with the t-test with corrected *p*-values, a trend was still present. The absence of an effect of emotion on item memory is concerning because it could suggest that participants did not experience our stimuli as emotional. However, our images were rated as highly negative in a previous study (*M* = 2.75 on a scale of 1-negative to 9-positive) and arousing (*M* = 6.9 on a scale of 1-relaxed to 9-aroused; Riberto et al., 2022). One possible explanation for the lack of emotional enhancement on recognition memory is the control of our stimuli for thematic similarity. Previous studies suggested that emotion-related differences in recognition accuracy may be due to the greater semantic closeness of emotional stimuli compared to neutral ones (Dougal & Rotello, 2007; Maratos et al., 2000; Sommer et al., 2008 but see Choi et al., 2013). One study first reported reduced recognition accuracy for emotional words compared to neutral, yet when words were controlled for semantic relatedness, no emotional effect was found (Dougal & Rotello, 2007). In current experiments, although recognition memory accuracy was not impacted by emotion, non-registered, exploratory analysis still showed an increased proportion of Hits in the negative condition compared to neutral condition. A difference in the BR index was also found. Differences in Hits and BR indices between the two conditions suggest an increased liberal bias for the negative condition compared to the neutral one, although BR indices of both conditions were in the conservative range.

It is also important to note that several studies previously reported no effect of emotion on recognition memory even if an effect was found for other types of memory such as subjective recollection or associative memory (Bisby et al., 2018; Sharot et al., 2004; Sharot & Yonelinas, 2008). Recognition memory measures may miss an effect of emotion because emotion influences recollection, not familiarity; but here emotion also did not influence the proportion of “Remember” responses. Again, this null effect contrasts with previous literature (Dewhurst & Parry, 2000; Kensinger & Corkin, 2003; Rimmele et al., 2011, 2012; Sharot et al., 2004), but this finding may be due to immediate retrieval tests. Indeed, there is evidence that the emotional effect on subjective recollection is time-dependent (Sharot et al., 2007; Sharot & Yonelinas, 2008; for a review see; Yonelinas & Ritchey, 2015).

One concern may be the online setting of the current experiment which may have impacted the results. Importantly, both experiments included strong attentional checks ensuring that participants that were not paying attention were excluded from the final dataset. We note, however, that some previous in-person experiments reported higher recognition accuracy (Hits ranged between 0.9 and 0.77; Bisby et al., 2018; D’Argembeau & Van der Linden, 2005; Palombo et al., 2020; Rimmele et al., 2012) compared to our study (Hits_neut _= 0.726; Hits_neg _= 0.793). Nonetheless, comparable levels of performance to our study are found in other previous in-person studies (Guez et al., 2015; Maddox et al., 2012; Murray & Kensinger, 2012; Touryan et al., 2007; when words as stimuli). Importantly, we believe that the slightly lower, albeit well above-chance recognition memory performance may be due to the tighter control of our stimuli rather than the online setting. To control for semantic similarity, our images represented only four types of scenes, rendering lures more similar to targets.

Mixed effect models were generated to analyse whether temporal and contextual memory were more accurate for images that were Remembered as compared to Known, but it was not the case. This contrasts with Ventura-Bort and colleagues’ study (2020). In that study, participants saw neutral objects embedded in emotional or neutral scenes. One week later, the retrieval task required participants to undertake a Remember/Know paradigm for the neutral object and an associative memory task asking whether the object was paired with a pleasant or unpleasant scene. Overall, no difference between negative and neutral conditions was found for the number of Remember responses for the object, but for correct associative trials only, more Remember responses were given, and this effect was stronger for negative scenes. These findings suggest that accurate contextual memory is driven by subjective recollection. The discrepant results may be because in our study the context was neutral and the images emotional, whereas the inverse was used in their paradigm. Similar to our study, other evidence showed that increased subjective recollection is not necessarily linked to increased associative memory for contextual details. Rimmele and colleagues (2011) reported a double dissociation between subjective recollection and contextual memory; despite increased Remember responses for emotional scenes, memory for contextual details, such as frame colour (Experiment 1) or unrelated objects paired with scenes (Experiment 2), was decreased for emotional scenes, even for those that received a Remember response. In the same vein, Sharot and Yonelinas (2008) reported increased subjective recollection for emotional compared to neutral images after 24 h delay, but no difference in contextual memory accuracy for a task completed while encoding the images. Nonetheless, interestingly, Rimmele and colleagues (2012) found that subjective recollection was accompanied by better associative memory for the location on the screen and the block of the images. The mixed findings may be due to the type of contextual detail tested; it seems that details intrinsically related to items (blocks, location in the screen, scene-type; pleasant/unpleasant) are related to subjective recollection whereas details extrinsically related to items (task completed while viewing items; unrelated paired objects; frame colour) are not linked to subjective recollection. This plausible explanation is in line with the object-based theory (Mather, 2007), where intrinsic emotional items features are strongly bound and remembered, but memory for the associations between emotional items and other items or the context is reduced, suggesting a different emotional impact on distinctive details tested.

In addition, partially consistent with the object-based theory, our results show that emotion decreased contextual memory but not temporal source memory. Temporal source is more intrinsic to the emotional images participants encoded here, compared to the paragraphs images were interleaved with. Therefore, the disparate influence of emotion on these two memory tests lends indirect support for the object-based account.

The object-based account suggests that when a unitization process is applied during encoding, contextual memory for the emotional condition is improved. Indeed, with unitization instructions, the context becomes part of the item and no longer relies on hippocampal-based associative memory but on item-memory (Han et al., 2018; Murray & Kensinger, 2013). This account therefore suggests that unitization is a key component for subsequent associative memory accuracy. In the present experiments, we did not manipulate unitization, but we asked participants to rate their ability to integrate the images and the texts together. Although participants found it difficult to integrate images and paragraphs, evident in low integration ratings, ability to integrate was still lower in blocks containing negative images, replicating previous findings (Caplan et al., 2019). Poorer integration of images and paragraphs in these blocks may explain the decrease in contextual memory performance in our study. Reduced subjective integration during encoding of emotional compared to neutral pairs has been suggested as a potential cause of subsequent decreased associative memory for emotional pairs (Caplan et al., 2019). Nonetheless, regression analyses (reported in the Appendix) showed that integration ratings did not correlate with memory performance. The hypothesis that integration may play a role in subsequent associative memory should be further explored in studies where it is easier to integrate the emotional target items with their context. Such work could manipulate integration intention and add an objective measure of integration beyond ratings. Overall, the poorer integration found for negative blocks suggests that reduction of integration during an emotional event could play a role during an intensely emotional situation such as a trauma. This assumption aligns well with the dual representation account’s claim that within-event hippocampal binding is disrupted during a traumatic situation and is related to PTSD symptoms development.

With this study, we also wanted to assess whether some types of associative memory are related. The mixed effects models of Experiments 1 and 2 showed that temporal source and contextual memory are related, as contextual memory was a significant predictor when temporal source memory was the dependent variable and vice versa. These results suggest that memory tests assessing temporal source and contextual memory may be partially measuring the same mechanisms. Temporal source memory tests the ability to retrieve block identity; although one could use memory for the contextual details of the block (the story) to deduce the temporal source of an item, the task could also be solved only with memory for temporal aspects. In contrast, contextual memory requires participants to retrieve contextual details: they could do so with no memory of the temporal aspects of the situation. Nonetheless, mixed effect models showed that these two types of memory are related. In our task, when participants were asked to retrieve the context of an image, the temporal aspect and other aspects may be retrieved together. The fact that we observed this correlation even when controlling for random effects of participants suggests that they are not due to effects such as the level of motivation of each participant. The current paradigm was designed such that each block has a unique temporal context as well as a shared context (i.e. topic of the text), a common feature of daily life, where episodes which are close in time often share similar spatial or semantic contexts (eating and doing the dishes happen close in time and in similar contexts, such as the kitchen, taking a shower and getting dressed in the bathroom etc). Nevertheless, this aspect of the task may have favoured the relationship between the two types of memory. More studies are needed to explore the relationship between contextual memory and temporal source memory using different paradigms, for example where the same content (e.g. an image) is paired with multiple items.

Lastly, block-type appears to be a significant predictor for temporal source memory (Experiment 1 but not Experiment 2) and contextual memory (Experiment 2 and Experiment 1 and 2 combined, but not Experiment 1), revealing the same effect: memory was better for pure compared to mixed blocks. This effect suggests that both types of associative memory are impacted by similar contextual features, again implying that they rely partially on the same mechanisms. Future studies could investigate it further. Interestingly, an effect of block-type was also found for subjective recollection, but in the opposite direction: increased Remember responses for mixed blocks compared to pure blocks. This effect may be due to increased image distinctiveness in mixed compared to pure blocks: in mixed blocks, the images may appear more distinctive from each other as they differ in valence and in their content, and this may help subjective recollection. Dewhurst and Parry (2000) found similar results using words as stimuli: when subjective recollection was tested in mixed lists, an emotional effect was found showing increased Remember responses, but when it was tested for pure lists, no emotional effect was seen. Researchers also suggested that an effect of distinctiveness may explain their results. Finally, it is relevant to see that block-type has opposite effect on distinct types of associative memory (subjective recollection vs contextual and temporal source memory), suggesting that they rely on different mechanisms.

One potential limitation of the current study is the small number of trials, which could decrease the power of the study (Baker et al., 2021). The current paradigm represents a compromise between paired-associate, list-ordering and source memory paradigms, which all use different numbers of trials. Our aim to be able to test multiple aspects of associative memory together meant that the paradigm represents a compromise between these traditions. It was not possible to increase trial numbers in our paradigm because reading texts, which nicely implements the unfolding of time, is attention- and time-consuming, and the quality of data may be decreased when studies, especially online studies, are too long.

In addition, soliciting memory for the timing of each image within the block allowed us to obtain a number of measures of order memory for each block, which is an advantage over list reconstruction methods where only a single score is typically afforded, and which therefore forces experimenters to present a large number of lists (Clewett & McClay, 2021, p. 16 lists; DuBrow & Davachi, 2013, p. 16 lists; Serra & Nairne, 1993, p. 24 lists). However, it is worth noting that the order memory accuracy for each trial within each block may be correlated, potentially decreasing power.

Although the number of trials here is smaller compared to previous studies, it is compensated by sample sizes that are considerably larger than in previous studies with N ranging from 17 to 35 (Bisby et al., 2018; Madan et al., 2017; Rimmele et al., 2012; Schmidt et al., 2011; Sharot et al., 2007; Sharot & Yonelinas, 2008). Statistically, the surest way to increase power is to increase sample size. Increasing the number of trials can increase power only if the variability of the effect in the population is small relative to the trial-by-trial variability of the effect within participants, which is not always the case and is difficult to be assessed prior to data collection (Rouder & Haaf, 2018).

To conclude, our study partly supports the dual representation account because across all the different types of associative memory we have tested, emotion decreased integration as well as contextual memory. This result aligns with the hypothesis that highly emotional situations would induce weaker hippocampal-dependent associative binding between items and their surrounding non-temporal context. Poorer binding of the trauma and its past context may facilitate involuntary retrieval of trauma content in trauma-unrelated contexts. However, in contrast to the further hypothesis of the dual representation account that memory for both spatial and temporal trauma context will be impaired, we did not observe an effect of emotion on temporal memory. It is worth noting that although the current paradigm examines the impact of emotion on associative memory, it is not a PTSD model. Therefore, no direct conclusion drawn from these results can be applied to a PTSD condition. Furthermore, our results clearly challenge theories that argue that emotion mandatorily increases the association between items and their context. Here, we did not observe evidence either for strong binding between the neutral context and the emotional event, for increased subjective recollection for emotional items, or for increased temporal source or order memory. Our results leave open the possibility that items are more strongly associated to their context when the context itself is emotional. eCMR and CMR3, indeed, suggest that emotion increases binding of an emotional item to its emotional, but not temporal context. This possibility deserves further research.

## Data Availability

https://osf.io/scqyn/?view_only=fc3c78bd18414feda57c0486a3a67f51.

## Appendix

To test to what extent emotion (neutral, negative), block-type (mixed, pure neutral, pure negative), integration ratings as well as the different memory performance at each test contribute to memory performance for each test, multiple linear regressions across blocks were calculated with a stepwise procedure. Assumptions for multiple regressions were tested and met.

### Experiment 1

1st regression:

*Order memory = Emotion + Block-type + Integration ratings + Contextual memory + Temporal source memory*.

For order memory performance as the dependent variable, no model was significant (*F*(5, 222) = 1.518, *p = 0.185* with an *R*^2^ of .033; not included in the Table A1).

2nd regression:

*Contextual memory = Emotion + Block-type + Integration ratings + Order memory + Temporal source memory*.

For contextual source memory as the dependent variable, the only model that is significant includes temporal source memory performance as a predictor (*F*(1,226) = 82.7, *p *< .001 with an *R*^2^ of .268).

3rd regression:

*Temporal source memory = Emotion + Block-type + Integration ratings + Order memory + Contextual memory*.

For temporal source memory as the dependent variable, the first significant regression equation includes only the contextual memory performance as the predictor (*F*(1,226) = 82.7, *p *< .001 with an *R*^2^ of .268). The second significant regression equation also includes block-type as a predictor (*F*(2,225) = 46.466, *p *< .001 with an *R*^2^ of .292).

### Experiment 2

1st regression:

*Contextual memory = Emotion + Block-type + Integration ratings + Recognition memory + Remember response + Temporal source memory*.

For contextual memory as the dependent variable, the first significant regression equation includes only temporal source memory performance as the predictor (*F*(1, 245) = 221.561, *p < 0.001* with an *R*^2^ of .475). The second significant regression equation also includes block-type as a predictor (*F*(2, 244) = 119.407, *p < 0.001* with an *R*^2^ of .495).

2nd regression:

*Temporal source memory = Emotion + Block-type + Integration ratings + Recognition memory + Remember response + Contextual memory.*

For temporal source memory as the dependent variable, the model found to be significant includes contextual memory as the only predictor (*F*(1, 245) = 221.561, *p < 0.001* with an *R*^2^ of .475).

3rd regression:

*Recognition memory = Emotion + Block-type + Integration ratings + Remember responses + Temporal source memory + Contextual memory*.

For recognition memory as the dependent variable, the model found to be significant includes the subjective feeling of Remembering as the only predictor (*F*(1, 245) = 80.644, *p < 0.001* with an *R*^2^ of .248).

4th regression:

*Remember responses = Emotion + Block-type + Integration ratings + Recognition memory + Temporal source memory + Contextual memory.*

For remember responses as the dependent variable, the model that was found to be significant includes recognition memory as the only predictor (*F*(1, 245) = 80.644, *p < 0.001* with an *R*^2^ of .248).

**Table A1.** Regression models for Experiment 1.

Dependent variable	Models	Intercept and predictors	Standardized coefficients beta	*t*	Sig.
Contextual memory	1	Intercept		6.322	<.001
		Temporal source memory	.518	9.094	<.001
Temporal memory	1	Intercept		9.567	<.001
		Contextual memory	.518	9.094	<.001
	2	Intercept		9.839	<.001
		Contextual memory	.490	8.605	<.001
		Block-type	−.159	−2.785	.006

**Table A2.** Regression models for Experiment 2.

Dependent variable	Models	Intercept and predictors	Standardized coefficients beta	*t*	Sig.
Contextual memory	1	Intercept		8.022	<.001
		Temporal source memory	.689	14.885	<.001
	2	Intercept		8.609	<.001
		Temporal source memory	.675	14.760	<.001
		Block-type	−.141	−3.088	.002
Temporal memory	1	Intercept		4.021	<.001
		Contextual memory	.689	14.885	<.001
Recognition memory	1	Intercept		25.941	<.001
		Remember responses	.498	8.980	<.001
Remember responses	1	Intercept		−.983	.326
		Recognition memory	.498	8.980	<.001

**Table A3.** Demographics of the participants.

		Experiment 1	Experiment 2
Race	White	50%	56.1%
	Black	28.8%	29.3%
	Asian	13.7%	7.3%
	Mixed	6.8%	7.3%
Country of residence	UK	45.2%	47%
	South Africa	28.7%	36.1%
	USA	5.5%	6%
	Australia	5.5%	0%
	Ireland	5.5%	2.4%
	Canada	4.1%	3.6%
	New Zealand	2.7%	1.2%
	Spain	1.4%	1.2%
	Netherland	1.4%	1.2%
	Italy	0%	1.2%
Nationality	UK	43.8%	44.6%
	South Africa	27.4%	33.7%
	Australia	6.8%	0%
	Ireland	5.5%	3.6%
	USA	4.1%	6%
	Canada	4.1%	4.8%
	New Zealand	2.7%	1.2%
	Nigeria	1.4%	1.2%
	Algeria	1.4%	0%
	Jamaica	1.4%	0%
	Zimbabwe	1.4%	2.4%
	Italy	0%	1.2%
	Sweden	0%	1.2%

**Table A4.** Generalised linear mixed effect models of Experiment 1.

Dependent variable	Intercept and predictors	Estimate	Standard Error	*Z* value	Pr (>|z|)
Order memory	Intercept	1.314	0.147	8.895	<0.001
	Contextual memory	−0.141	0.075	−1.880	0.0602
	Temporal source memory	0.023	0.074	0.310	0.756
	Emotion	0.107	0.069	1.535	0.124
	Block-type	−0.092	0.067	−1.370	0.170
	Contextual memory: Emotion	−0.020	0.073	−0.272	0.785
	Temporal source memory: Emotion	−0.002	0.073	−0.034	0.972
Temporal source memory	Intercept	0.403	0.085	4.717	<0.001
	Order memory	0.016	0.072	0.232	0.816
	Contextual memory	−0.630	0.062	−10.087	<0.001
	Emotion	−0.093	0.070	−1.322	0.186
	Block-type	0.215	0.060	3.581	<0.001
	Order memory: Emotion	0.003	0.070	0.052	0.958
	Contextual memory: Emotion	−0.238	0.061	−3.856	<0.001
Contextual memory	Intercept	0.404	0.102	3.959	<0.001
	Order memory	−0.151	0.074	−2.039	0.0414
	Temporal source memory	−0.623	0.063	−9.807	<0.001
	Emotion	0.072	0.071	1.012	0.311
	Block-type	0.160	0.061	2.601	0.009
	Order memory: Emotion	−0.042	0.071	−0.596	0.551
	Temporal source memory: Emotion	−0.248	0.062	−3.968	<0.001

**Table A5.** Generalised linear mixed effect models of Experiment 2.

Dependent variable	Intercept and predictors	Estimate	Standard error	*Z* value	Pr (>|z|)
Remember responses	Intercept	0.455	0.282	1.615	0.106
	Contextual memory	0.007	0.106	0.074	0.941
	Temporal memory	−0.144	0.105	−1.378	0.168
	Emotion	−0.040	0.078	−0.522	0.601
	Block-type	0.176	0.076	2.306	0.021
	Contextual memory: Emotion	−0.006	0.104	−0.061	0.951
	Temporal memory: Emotion	−0.050	0.102	−0.498	0.618
Temporal source memory	Intercept	−0.240	0.116	−2.058	0.039
	Remember responses	−0.110	0.092	−1.192	0.233
	Contextual memory	−1.767	0.095	−18.439	<0.001
	Emotion	−0.060	0.086	0.702	0.482
	Block-type	−0.135	0.083	−1.620	0.105
	Remember responses: Emotion	−0.051	0.084	−0.612	0.540
	Contextual memory: Emotion	0.151	0.086	1.749	0.080
Contextual memory	Intercept	0.509	0.115	4.409	<0.001
	Remember responses	−0.009	0.093	−0.097	0.922
	Temporal source memory	−1.762	0.095	−18.456	<0.001
	Emotion	0.119	0.087	1.374	0.169
	Block-type	0.350	0.085	4.101	<0.001
	Remember responses: Emotion	−0.058	0.085	−0.691	0.489
	Temporal source memory: Emotion	0.183	0.086	2.107	0.035

**Table A6.** Generalised linear mixed effect models of Experiments 1 and 2.

Dependent variable	Intercept and predictors	Estimate	Standard Error	Z value	Pr (>|z|)
Temporal source memory	Intercept	0.135	0.062	2.164	0.030
	Contextual memory	−1.064	0.049	−21.560	<0.001
	Emotion	−0.029	0.047	−0.621	0.534
	Block-type	0.085	0.046	1.813	0.069
	Contextual memory: Emotion	−0.080	0.048	−1.672	0.094
Contextual memory	Intercept	0.404	0.066	6.101	<0.001
	Temporal source memory	−1.065	0.049	−21.509	<0.001
	Emotion	0.155	0.047	3.237	0.001
	Block-type	0.182	0.047	3.817	<0.001
	Temporal source memory: Emotion	−0.079	0.048	−1.642	0.100

**Table A7.** Images for sets 1 and 2 were controlled for luminance, contrast, Red, Green, Blue, Jpeg, Entropy, valence and arousal.

	Categories				Main effect						Interaction							
					Emotion		Category		Set		Category* Emotion		Emotion* Set		Category* Set		Category* Set* Emotion	
	Poverty	Car accident	Laundry	Phone	*F*	*p*	*F*	*p*	*F*	*P*	*F*	*P*	*F*	*p*	*F*	*p*	*F*	*p*
Luminance Set A	113.57 ± 21.75	106.64 ± 33.44	107.37 ± 28.01	102.13 ± 28.01	1.5	>.10	<1	>.10	<1	>.10	<1	>.10	2.32	>.10	<1	>.10	<1	>.10
Luminance Set B	105.65 ± 20.99	99.293 ± 11.97	117.115 ± 33.37	107.388 ± 34.264														
Contrast Set A	55.12 ± 9.311	62.16 ± 8.14	60.45 ± 10.24	59.14 ± 2.5	<1	>.10	<1	>.10	3.29	>.10	1.24	>.10	<1	>.10	<1	>.10	<1	>.10
Contrast Set B	61.77 ± 11.43	65.84 ± 4.37	66.51 ± 13.65	65.14 ± 17.69														
R Set A	122.67 ± 21.577	107.45 ± 32.98	115.24 ± 29.05	104.32 ± 22.11	<1	>.10	3.1	>.10	<1	>.10	<1	>.10	1.1	>.10	<1	>.10	<1	>.10
R Set B	115.56 ± 18.39	105.52 ± 19.22	130.98 ± 30.72	112.35 ± 35.27														
G Set A	111.39 ± 22.46	105.06 ± 33.69	105.36 ± 28.73	101.36 ± 26.78	<1	>.10	<1	>.10	<1	>.10	<1	>.10	<1	>.10	<1	>.10	<1	>.10
G Set B	102.02 ± 22.07	96.77 ± 9.6	112.92 ± 35.79	106.36 ± 33.37														
B Set A	100.89 ± 22.30	112.64 ± 35.03	97.123 ± 29.07	100.44 ± 27.78	<1	>.10	<1	>.10	<1	>.10	<1	>.10	<1	>.10	<1	>.10	<1	>.10
B Set B	98.33 ± 25.95	95.84 ± 6.35	102.35 ± 32.15	99.53 ± 40.93														
Jpeg Set A	72879 ± 8016	58022 ± 8988	62750 ± 15649	71843 ± 36137	<1	>.10	<1	>.10	<1	>.10	2.246	>.10	<1	>.10	1.65	>.10	<1	>.10
Jpeg Set B	61717 ±10389	68407 ±14077	53886 ± 4149	70277 ± 31706														
Entropy Set A	7.55 ± .26	7.57 ± .22	7.64 ± .21	7.51 ± .24	<1	>.10	<1	>.10	<1	>.10	<1	>.10	<1	>.10	<1	>.10	<1	>.10
Entropy Set B	7.66 ± .17	7.67 ± .17	7.47 ± .2	7.50 ± .28														
Valence Set A	2.65 ± .207	1.66 ± .35	5.03 ± .265	5.05 ± .234	838.79	<.001	22.88	<.001	<1	>.10	36.01	<.001	1.27	>.10	<1	>.10	<1	>.10
Valence Set B	2.85 ± .463	1.76 ± .314	4.866 ± .467	5.08 ± .24														
Arousal Set A	6.95 ± .535	7.866 ± .242	4.61 ± .248	4.68 ± .132	922.3	<.001	36.0	<.001	<1	>.10	22.26	<.001	<1	>.10	<1	>.10	<1	>.10
Arousal Set B	6.86 ± .480	7.90 ± .260	4.53 ± .242	4.7 ± .109

**Table A8.** Reading time in ms for each text.

	Mean	Std. Deviation	Std. Error
Text 1	83006.79	82660.28	7545.82
Text 2	79380.70	74927.32	6839.9
Text 3	63221.84	55566.20	5072.48
Text 4	75655.58	73503.95	6709.96
